# Characterization of a novel pulse normalization technology for beam scanning of small fields without a reference chamber

**DOI:** 10.1002/acm2.14379

**Published:** 2024-05-03

**Authors:** Garrett C. Baltz, Steven M. Kirsner

**Affiliations:** ^1^ Scripps Cancer Center San Diego California USA

**Keywords:** beam scanning, commissioning, pulse normalization, relative dosimetry, small field, stereotactic

## Abstract

**Purpose:**

A novel pulse normalization technology enabling the acquisition of low noise beam data without the use of a physical reference chamber has recently been commercially released. The purpose of this study was to characterize the use of this technology for beam scanning of small fields required in the commissioning of a stereotactic radiotherapy program.

**Methods:**

Three detectors (Edge diode, microDiamond, PinPoint) were used to acquire beam data under three conditions: with a reference chamber, with pulse normalization and no reference chamber (PN), and without pulse normalization and no reference chamber (nPN). Percent depth dose (PDD) scans were acquired for 0.5, 1.0, 2.0, and 3.0 cm^2^ field sizes and profiles were acquired at 1.4, 10, and 30 cm depths using continuous scanning. The coefficient of variation (CoV) was calculated for all beam data to compare signal‐to‐noise and gamma comparisons (1%, 1 mm) were calculated of the PN and nPN scans compared to the reference data.

**Results:**

Average 95th percentile CoV values were similar for all detectors across conditions, with PN data being comparable to reference data and minor increases observed for nPN data. Mean gamma pass rates for PN PDD scans exceeded 98% for all detectors. Profile gamma pass rates were 100% for all detectors at 1.4 and 10 cm depth. At 30 cm depth, profiles acquired with the PinPoint and microDiamond detectors had lower mean gamma pass rates than the Edge, at 95% and 95.7%, respectively.

**Conclusions:**

A novel pulse normalization technology was demonstrated to be effective for acquiring beam profiles and PDDs for small fields without the use of a physical reference chamber. Limitations in how the method is implemented led to some errors in data acquired using lower sensitivity detectors. When used with a diode, pulse normalization produced equivalent scans to those acquired with a reference chamber.

## 1 INTRODUCTION

Relative dosimetry of small fields is one of the primary challenges when commissioning a stereotactic radiotherapy program. Small‐field dosimetry measurements are subject to conditions of loss of lateral charged particle equilibrium, and their small size requires the use of specialized detectors capable of acquiring accurate measurements of the field profile. Best practices for small‐field dosimetry have been published in multiple scientific reports by both the AAPM and IAEA.^1^, ^2^ Most literature on dosimetry of small fields has focused on the challenges of measuring output factors for small‐fields due to the change in detector response versus field size.^3^, ^4^ While this is a major factor to consider, there are also challenges when acquiring beam profiles and percent depth dose (PDD) data.

When acquiring beam data, AAPM TG‐106 recommends the use of a reference detector to correct for instantaneous drifts or fluctuations in the beam output.^5^ This can present challenges when collecting beam data for small‐fields required by specialized stereotactic treatment planning systems (TPS),^6^ as the reference detector may occupy a significant portion of the primary radiation field when scanning field sizes of 2 × 2 cm^2^ or smaller.^7^ Indeed, for small fields AAPM TG‐155 recommends that a reference detector not be used due to possible perturbations, and that instead a scan be acquired either without a reference chamber in a step‐wise time integration technique, or with the reference detector placed at the bottom of the water tank.^1^ The integration method, while effective, represents a significant time penalty compared to continuous scanning, while the second method is specifically recommended against by TG‐106, as the reference detector may become loose while submerged and produce inconsistent results.^5^ In addition, depending on the scanning system used, when the reference detector is placed at the bottom of the tank there is a significant likelihood that field chamber will pass in the path of the reference detector during scanning, causing the reference signal to change leading to bad quality beam data. Transmission reference detectors are also a possible solution. However, aside from any possible perturbation of the beam, many scanning systems do not allow for setting a field diode at no applied voltage bias while also using a reference ionization chamber with an applied high voltage bias. It is clear from the published literature there are many limitations related to the use of a reference chamber that must be considered when acquiring beam data for small fields.

A scanning phantom manufacturer, Sun Nuclear (Melbourne, Florida), has recently commercially released a new scanning system that is equipped with a pulse normalization technology to overcome the limitations associated with placing a reference detector within the field for small‐field measurements. Pulse normalization allows for the acquisition of beam data with minimal noise by normalizing the measured signal using the number of beam pulses acquired during the acquisition in lieu of a reference chamber signal. This technology allows for the scanning of small fields utilizing a continuous mode scanning technique without the use of a reference chamber. The purpose of this study was to characterize the use of this novel pulse normalization technology for the acquisition of beam profiles and PDDs in small‐field dosimetry conditions.

## 2 METHODS AND MATERIALS

### 2.1 Scanning hardware and detectors

All beam data were acquired using the Sun Nuclear SunSCAN 3D system. This system consists of a cylindrical water phantom with integrated positioning motors and scanning arms capable of 0.1 mm positioning accuracy. The included electrometer unit can capture charge with a resolution of 1 µs. When used with the accompanying Sun Nuclear SunDOSE software, the system can acquire normalized beam data using what the manufacturer calls a “virtual reference detector” via the pulse normalization method.

**FIGURE 1:**
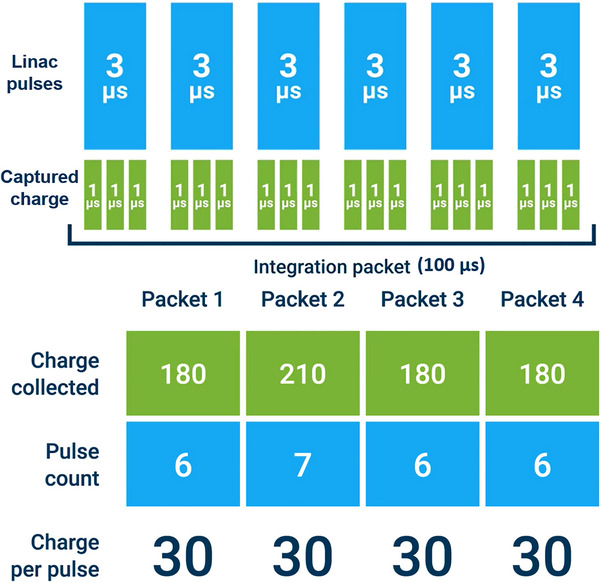
Diagram of the pulse normalization method as implemented in the SunSCAN system.

A diagram detailing how the pulse normalization method works is presented in Figure 1. Throughout data collection, the electrometer collects charge in 1 µs intervals, which allows it to discriminate individual beam pulses. Every 100 µs, an integrated charge packet is sent to the SunDOSE scanning software. The software divides the total charge collected by the number of pulses contained in the integration packet to determine the charge per pulse, which in turn is used to normalize the readings.

In order to test the robustness of the pulse normalization method based on detector type, three different types of detectors commonly used for small‐field dosimetry were studied. They included the Sun Nuclear Edge Diode, PTW microDiamond (PTW Dosimetry, Freiburg, Germany), and the PTW PinPoint Ionization chamber. The detectors and their properties are presented in Table 1.

**TABLE 1:** Summary of detectors used and their properties.

Detector	Type	Detector volume (cm^3^)	Detector diameter (mm)	Sensitivity (nC/Gy)
Sun Nuclear Edge	Diode	1.9E‐05	0.8	32
PTW micro Diamond 60019	Diamond	4E‐06	2.2	1.0
PTW Pin Point TN31006	Ionization chamber	0.015	7.0	0.4

### 2.2 Beam scanning

All profile and PDD scans were acquired from a Varian TrueBeam STx (Varian Medical Systems, Palo Alto, California) linear accelerator using the 6X‐FFF photon beam. TG‐155 recommends small field considerations be used when the field size is 3 × 3 cm^2^ and smaller.^1^ Therefore, the current study used MLC defined field sizes of 3 × 3, 2 × 2, 1 × 1, and 0.5 × 0.5 cm^2^ (with the jaws set 0.6 cm larger than the MLC defined field), which are common field sizes required in the commissioning of stereotactic radiosurgery specific treatment planning systems such as Brainlab Elements (Brainlab, Munich, Germany). Beam profiles (crossline and inline) were acquired at depths of 1.4, 10, and 30 cm, and PDDs were acquired down to 30 cm depth for all field sizes.

**FIGURE 2:**
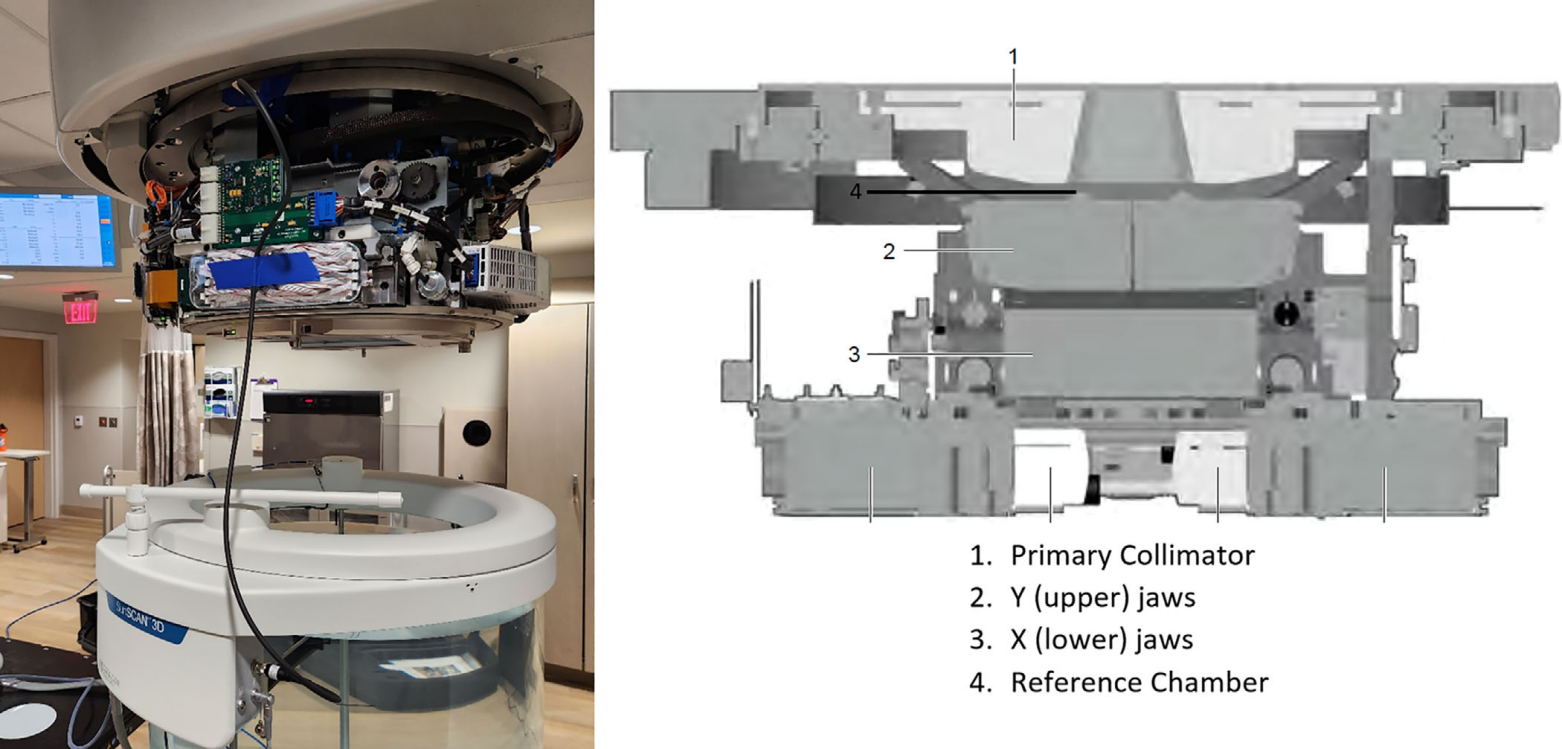
Location of the reference detector in the head of the linear accelerator below the primary collimator.

To compare scans acquired with pulse normalization to scans acquired using conventional beam scanning techniques, scans were acquired under three different conditions. The first was with a reference chamber placed in the field within the head of the linear accelerator such that it would not interfere with the primary beam.^7^ This was accomplished by placing the reference detector just inside the beam under the primary collimator as marked by location 4 in Figure 2. The second condition was with no reference detector and pulse normalization on (PN), and the third condition was no reference detector with pulse normalization turned off (nPN).

A key assumption the pulse normalization method relies on to normalize the collected charge is that the dose per pulse is consistent. Varian linear accelerators can maintain the nominal dose rate of the beam by dynamically adjusting the pulse width, which can cause the dose per pulse to vary. Because of the potential of varying dose per pulse to negatively affect the efficacy of pulse normalization, it was recommended by the manufacturer to turn off the dose servos when acquiring beam data. All data were acquired with the beam at a nominal dose rate of 600 MU/min with the dose servos turned off. A lower dose rate than the maximum of 1400 MU/min for 6XFFF was used due to extended beam on time, however, as the dose per pulse for Varian accelerators is the same for all dose rates, it is not expected using a higher rep rate will alter the results of this study. Another customizable setting when using pulse normalization is the pulse detection threshold, which is a unitless value defining the difference in counts used by the electrometer to determine the occurrence of a pulse. The three detectors used in this study have different sensitivities, which means the counts collected per pulse will be different for each detector. Recommendations from the manufacturer as well as trial and error were used to determine the optimal pulse detection threshold for each detector. The pulse detection thresholds used were 232, 72, and 40 for the Edge, microDiamond, and PinPoint detectors, respectively.

Data were acquired using a continuous scanning technique with the detector moving at 0.1 cm/s. The scanning software automatically applies a median filter to the raw data, as recommended by AAPM TG‐155.^1^ The median filter was consistently applied to all scans.

### 2.3 Data analysis

For data analysis, scans were exported from the Sun Nuclear Dosimetry software with a grid size of 0.01 cm. A script developed in Python v2.8 was used to calculate the rolling coefficient of variation (CoV) of the scan, which was used to quantify the signal‐to‐noise ratio.^5^ CoV was calculated by dividing the rolling standard deviation by the rolling mean of the last 10 data points. The mean and 95^th^ percentile of the CoV was calculated within the 90% dose region of profiles, and Dmax and deeper for PDDs.

To assess if pulse normalization would adversely affect the integrity of the profiles or PDDs, gamma comparisons were performed for the PN and nPN scans compared to the reference scans. Gamma analysis was calculated using the Sun Nuclear Dosimetry software using 1% dose agreement (local normalization) and 1 mm distance to agreement criteria for all comparisons.

Statistical significance of differences in CoV for PN and nPN scans compared to reference, as well as the gamma pass rates of PN and nPN scans, was tested using a Mann‐Whitney *U* test with *α* = 0.05.

## 3 RESULTS

### 3.1 Signal to noise comparisons

**FIGURE 3:**
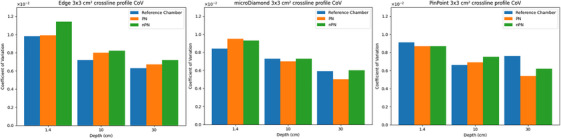
Bar plots of 95th percentile CoV for 3 × 3 cm^2^ crossline profiles for all detectors.

Plots comparing the 95^th^ percentile CoV for the 3 × 3 cm^2^ crossline profiles are shown in Figure 3. Larger 95^th^ percentile values correspond to the profile being noisier. For the Edge detector, the scans with pulse normalization had lower noise than the nPN scans, and slightly higher noise than the reference scans at all depths. The microDiamond detector demonstrated mixed results, with the PN scan noisier than the reference and nPN scans at 1.4 cm depth. However, for deeper depths the PN scan had lower noise than both the reference and nPN scans. Profiles acquired with the Pinpoint detector using PN showed equivalent or lower noise than the nPN profiles at all depths, and lower noise than the reference scan at 1.4 and 30 cm scan depths. A global trend observed in the data is that the 95^th^ percentile CoV for all detectors decreases as the scan depth of the profile increases. This was primarily due to the profile broadening with increasing depth, lowering the relative contribution of the profile shoulders increasing the CoV. The trends observed in the 3 × 3 cm^2^ crossline profiles were consistent for the inline profiles and other field sizes.

**FIGURE 4:**
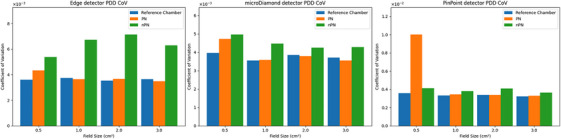
Bar plots of 95th percentile CoV for PDDs for all detectors.

**FIGURE 5:**
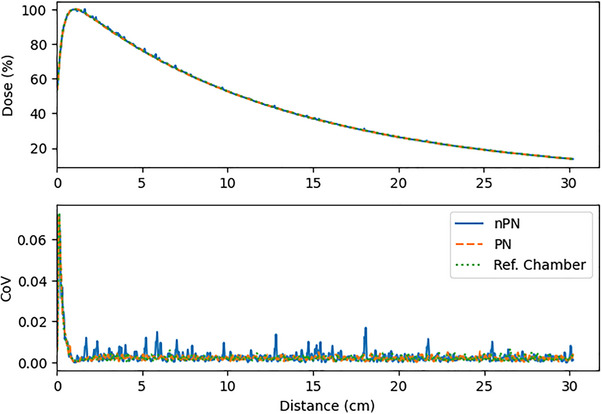
Example of noise spikes observed in Edge nPN PDD for 1 cm^2^ field size.

**FIGURE 6:**
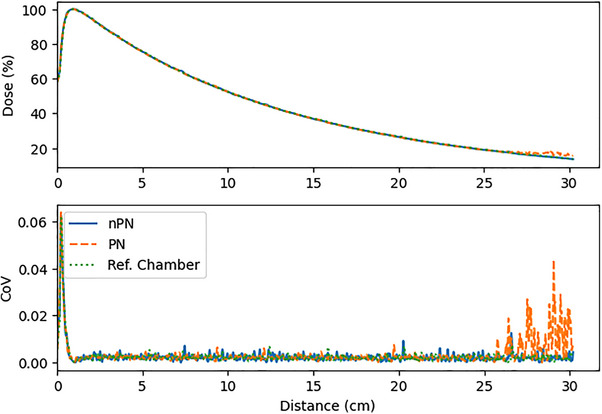
Noise observed in PinPoint PN PDD scan beyond 25 cm depth.

Results comparing the 95^th^ percentile CoV for the PDD scans are presented in Figure 4. For all detectors and field sizes, the PN scans had appreciably lower noise than the nPN scans. This was due to the presence of large noise spikes observed in the nPN PDD scans that were not present in the PN or reference chamber scans, as demonstrated in Figure 5. The magnitude of this effect was the largest for the Edge detector, but was also observed in the microDiamond scans, and minimally observed in the PinPoint scans. In general, for most PDDs the noise of the PN scan was comparable to the reference scan. One notable exception was the 0.5 cm^2^ field size PDD scan for the PinPoint detector, shown in Figure 6, which had a greater CoV compared to the reference and nPN scans due to the significant amount of noise that was present in the PDD at 25 cm depth and deeper. This effect was also observed for the microDiamond 0.5 cm^2^ field size PDD starting at 28 cm depth. The manufacturer was contacted regarding the observed noise in the PN scan, and the possible cause was thought to be related to the software behavior involving the detector signal. When the detector signal drops too low for the system to distinguish individual pulses, the software defaults to a previously determined nominal pulse count set during detector normalization (private communication). This transition can temporarily lead to inaccurate pulse counts being used to normalize the data. Due to the extremely low signal expected at 25 cm depth for a 0.5 cm^2^ field and the fact that the PinPoint chamber has the lowest sensitivity of the detectors studied, this software behavior appears to be the likely cause of the observed noise.

There were no statistical differences in CoV of the PN scans compared to reference for any of the detectors, with *p*‐values of 0.838, 0.851, and 0.980 for the Edge, microDiamond, and PinPoint, respectively. This was also the case for the CoV of the nPN scans, with *p*‐values of 0.800, 0.363, and 0.954, respectively.

### 3.2 Gamma analysis

Gamma pass rates for the PN and nPN scans compared to the reference scans for all detectors are presented in Table 2.

**TABLE 2:** Gamma pass rates for PN and nPN scans compared to reference scans.

			Gamma pass rate (%)							
		Field size (cm)	0.5		1.0		2.0		3.0	
		Depth (cm)	PN	nPN	PN	nPN	PN	nPN	PN	nPN
Edge	Crossline	1.4	100	100	100	100	100	100	100	100
		10	100	100	100	100	100	100	100	100
		30	100	100	100	100	100	98.55	100	100
	Inline	1.4	100	100	100	100	100	100	100	100
		10	100	100	100	100	100	100	100	100
		30	100	100	97.22	100	100	100	100	98.95
	PDD	–	99.51	99.18	100	98.68	99.67	97.69	99.67	97.03
microDiamond	Crossline	1.4	100	100	100	97.56	100	100	100	100
		10	100	100	100	100	100	100	100	98.78
		30	80.77	100	97.56	100	100	98.57	98.02	98.02
	Inline	1.4	100	100	100	100	100	100	100	100
		10	100	100	100	100	100	100	100	100
		30	95.65	100	100	100	93.85	100	100	95.88
	PDD	–	100	99.34	100	100	100	99.67	99.34	99.84
PinPoint	Crossline	1.4	100	100	100	100	100	100	100	100
		10	100	100	100	100	100	100	100	100
		30	88.89	100	88.37	100	100	100	100	99.03
	Inline	1.4	100	100	100	100	100	100	100	100
		10	100	100	100	100	100	100	100	100
		30	95.83	100	92.11	100	97.06	97.06	97.96	100
	PDD	–	92.26	100	100	99.84	100	99.84	100	100

Beam profiles acquired with the Edge detector using PN showed excellent agreement to the reference scans, with all but one scan having a pass rate of 100%. The nPN scans also had good overall agreement, with two scans lower than 100% pass rate due to the presence of noise in the profile. For the PDDs, the PN gamma pass rate was consistently higher than the nPN pass rate, due to the previously discussed noise spike phenomenon demonstrated in Figure 5.

**FIGURE 7:**
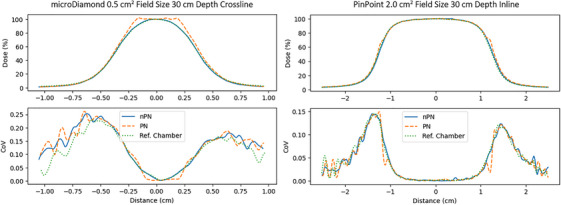
Left: clipping phenomenon observed for microDiamond PN scan for 0.5 cm^2^ field size at 30 cm depth. Right: Penumbra over‐response phenomenon observed for PinPoint PN profile.

For the microDiamond and PinPoint detectors, profiles with PN had 100% pass rates for all scans at 1.4 and 10 cm scan depths. Profiles acquired at 30 cm depth with PN had lower pass rates than the nPN scans. This was due to two separate issues observed in these scans, shown in Figure 7. The first observation was that the central in‐field portion of the profile would be “clipped off,” as if the detector signal had been saturated. This was observed for the 0.5 cm^2^ field size, 30 cm depth scans for the both the microDiamond and PinPoint. The second issue observed for larger field size scans at 30 cm depth was that the signal would abruptly over‐respond in the penumbra region, causing a discontinuity in this region. Both of these effects were observed in situations where the field signal would be very low. Consulting with the manufacturer, it seemed likely the previously discussed software behavior of switching to a nominal pulse count when the detector signal is too low was the cause for the observed behavior (private communication).

There was no statistical significance in the gamma pass rates of the PN and nPN scans for any of the detectors, with *p*‐values of 0.397, 0.779, and 0.202 for the Edge, microDiamond, and PinPoint detectors, respectively.

## 4 DISCUSSION

One of the primary aims of this study was to determine if pulse normalization could be used to acquire low noise beam data in small field size conditions using continuous scanning without the use of a reference detector. The results of this study indicate that pulse normalization was highly effective in this regard. In the case of beam profile scans, the PN scans had lower noise compared to the nPN scans and comparable noise to the reference scans for all detectors at all but one scan depth. When applied to acquiring PDD data, pulse normalization was highly effective in preventing noise spikes that were observed in the nPN scans. In addition to acquiring beam data with comparable noise to reference scan data, the results of the gamma comparisons show that when pulse normalization was functioning properly, it was not altering the integrity of the beam data and accurately represented the reference beam data.

A clear limitation of the manufacturer's currently implemented pulse normalization technique that was observed in this study was the anomalous data that was generated when the detector signal became too low. As currently implemented, when the detector signal becomes too low to discriminate pulses, the software will transition to using a nominal pulse count to normalize the signal. When in this transition period, it was observed this can cause an over response in penumbra regions or a saturation of the signal in the central part of the field. It is important to note that this phenomenon was only observed for scans at 30 cm depth for the microDiamond and PinPoint detectors, which have 32× and 80× lower sensitivity than the Edge diode detector, respectively. When using the Edge diode, the pulse normalization scans uniformly had lower noise than the nPN scans and all had a gamma pass rate of 99.5% or greater compared to reference scans. As diode detectors are one of the most commonly used detectors for acquiring beam data in small field conditions,^6^, ^8^, ^9^, ^10^, ^11^ pulse normalization was demonstrated to be a robust method to acquire low noise data without the use of a reference detector.

While it is always best practice to review beam data as they are acquired, based on the results of this study it is recommended extra attention be given when using PN with low sensitivity detectors to acquire profiles at deeper depths. However, this issue may be of minimal clinical relevance as profiles at 30 cm depth for these small of field sizes are not usually required for TPS commissioning. For example, Eclipse ignores beam data for any profiles smaller than 2 × 2 cm^2^ (Eclipse Photon and Electron Algorithms Reference Guide), and Brainlab only requires profiles down to 20 cm depth when commissioning the Monte Carlo algorithm (Brainlab RT Elements Technical Reference Guide). If data are needed in these conditions, a potential solution to prevent the issues observed would be to manually reduce the pulse detection threshold for these specific scans, rather than using the same threshold for all scans, although this was not tested in the current study. The manufacturer is aware of the limitations in the current software behavior and is working on improvements to automatically dynamically adjust the pulse detection threshold to prevent the irregular data (private communication).

An interesting observation in this study was that the majority of the no pulse normalization scans with no reference chamber had lower than expected noise and high gamma pass rates compared to the references scans. This can most likely be attributed to default application of the median filter to scans acquired in the Sun Nuclear Dosimetry software. When looking at the raw data, the median filter was able to smooth the majority of the noise in the nPN scans. One of the notable exceptions being for PDD scans, where it was not able to remove the large spikes in the data.

## 5 CONCLUSION

A novel pulse normalization technology capable of acting as a virtual reference chamber was found to be effective for acquiring beam profiles and PDDs for small field sizes without the need of a physical reference chamber. Beam data acquired using pulse normalization demonstrated high fidelity to reference scans and exhibited comparable signal‐to‐noise of data acquired using a reference chamber. In extremely low signal regions, limitations in how the pulse normalization method is currently implemented led to some errors in data acquired using lower sensitivity diamond detector and micro ionization chamber. When used with a diode detector, pulse normalization produced equivalent scans to those acquired with a reference chamber. This technology enables the ability to acquire low noise beam data for stereotactic field sizes using continuous scanning, which can reduce the time and effort required for commissioning.

## AUTHOR CONTRIBUTIONS

Garrett C. Baltz is responsible for the study design, acquisition and analysis of the data, and writing of the manuscript. Steven M. Kirsner is responsible for study design, acquisition and analysis of data, and editing of the manuscript.

## CONFLICT OF INTEREST STATEMENT

Scripps Cancer Center is a Sun Nuclear Patient Safety Center of Excellence and is a reference site for Sun Nuclear products.
